# Burden of risk factors for non-communicable diseases: an epidemiological review of the evidence from INDEPTH Health and Demographic Surveillance System (HDSS) in Indonesia

**DOI:** 10.1186/1471-2458-12-S2-A18

**Published:** 2012-11-27

**Authors:** Dwidjo Susilo, Istiti Kandarina, Siwi Padmawati, Laksono Trisnantoro

**Affiliations:** 1 Center for Health Service Management, Faculty of Medicine, Universitas Gadjah Mada, Yogyakarta 55281, Indonesia; 2 Study Program of Public Health, Faculty of Medicine and Health, Universitas Muhammadiyah Jakarta, Jalan KH Ahmad Dahlan, Ciputat, Jakarta 15419, Indonesia

## Background

Non-communicable diseases accounted for 64% of death in Indonesia at the end of 2008. Prevalence of tobacco use, unhealthy diet, and insufficient physical activities are quite high nationwide. It is also the case in Purworejo, a HDSS site in Indonesia. This study aims to review recent data in epidemiologic trends of risk factors for NCDs among adults (>18 years old) in Purworejo.

## Materials and methods

This is the first part of the ongoing INDEPTH Training and Research Centers of Excellence (INTREC) project funded by the EU, which included searching of INDEPTH-SAGE publications from 2001 through 2011 for the Purworejo HDSS site. This study reviewed published articles on the Purworejo HDSS.

## Results

Smoking prevalence among men in Purworejo increased from 54% in 2001 to 63% in 2005. While tobacco use was more prevalent in the older age groups of men ages 55-64 years old, the prevalence was almost non-existent among women in those years. It was noted in 2005, prevalence of overweight/obesity (BMI>25) in women was higher than in men (25% vs. 10%). This could be due to the fact that only 45% of women engaged in walking and cycling, compared to 75% of men who did the same activities. Women, furthermore, consumed fruit more often than men. Mean systolic blood pressure (SBP) among men (126.6 mmHg) was higher than women (123.4 mmHg) although the prevalence of hypertension among women (25%) was higher than men (24%).

## Conclusions

Men, older age and illiterate were significantly associated with high risk for the chronic NCDs while women, overweight/obese and older age were linked to the risk of hypertension. A comprehensive behavioral intervention and policy should be developed due to reduce the adverse effects of tobacco use, less physical activities and unhealthy diet on health of people in Purworejo.

